# Upon the Therapeutical Value of the Sulphites in Phlegmonous Angina

**Published:** 1870-09

**Authors:** Gerrard Geo. Tyrrell


*Upon the Therapeutical Value of the Sulphites in Phlegmonous Angina.*—Paper read before the Sacramento Society for Medical Improvement. By GERRARD GEO. TYRRELL, L.R.C.S.I., and K. & Q.C.P.I.

So much has been written, and so many conflicting opinions expressed, upon the therapeutical value of the sulphites in disease, that it is with the utmost diffidence I venture to bring the subject under the notice of the Society this evening. I am, however, emboldened to do so, from the conviction that I have discovered in the sulphites a certain prophylactic against, and a frequently successful agent in the cure of certain diseases affecting the throat, more especially in that common and most troublesome complaint known as phlegmonous angina, acute tonsillitis, or quinsy. I am still further stimulated to bring this subject under your observation from the fact that one of the great lights in medicine, whose eminent reputation is universal, and from whose teaching we have all acquired knowledge, the great Trousseau, says, "I

repeat, gentlemen, phlegmonous angina is one of those diseases which are alike the glory and despair of all medication: their despair, because medicine cannot prevail against them; we are powerless to check their progress, to abridge their duration. Their glory, because they cure themselves, whatever we may do; and the temptation is to ascribe to the medication the honor of the cure." He then sketches the rise and progress of the disease, alludes to the different treatment advised—incisions, leeching, cupping, bleeding, etc. — and says, in spite of all, the disease continues its progress; in four or five days, perhaps in nine or ten, an abscess forms, and bursts, and the patient is well; all the remedies have been useless, and the more violent injurious. "Expectation," he continues, "is the best treatment to follow in the disease which now occupies our attention. But this treatment, I admit, is most difficult to make acceptable in practice, especially when you are beginning your career, and have not yet inspired that confidence which you will do later. In order to respond to the just demands of your patients, prescribe remedies of no great power. If you really cannot cure them, at least leave some illusion to those who suffer, and do not make them despair by avowing your impotence; order acidulated and demulcent gargles and emollient fumigations, knowing perfectly well that they will effect nothing toward the cure of a disease which will in a short time come to a termination." Such is the language of Trousseau, and such his opinion of the incurability of acute tonsillitis. To moderate, if not entirely disprove, these very positive assertions, and to maintain the efficacy of treatment in anginose affections, will be my privilege this evening.

Although I am aware that authorities are not wanting who deny the therapeutical value of the sulphites in any disease, and even in this Society are there members whose scepticism upon this point is not disguised, I will endeavor to bring before your notice to-night the records of some cases in my own practice, no matter how feebly portrayed, which by their symptoms demonstrate the fact of either impending or established disease; and if I can satisfy you by such cases, treated after the manner hereinafter to be stated, that I have succeeded in arresting such disease, or even shortened its duration one day, then my object will have been accomplished, and your co-operation insured, in giving this agent a fair and impartial trial, and perhaps rescue from unmerited neglect a drug, powerful for good, and most worthy of our consideration. We are all conscious of the fact, although perhaps insensibly acquired, that the zymotic theory of disease has taken a firm hold upon our senses; and, indeed, reflection leads us to believe that many of the diseases which we were formerly willing to consider as local in their nature, and remedial by local treatment, are but the result of a certain poison or ferment in the blood, which evinces its presence by attacking certain organs, or producing certain effects, which are known to us under the names of diphtheria, erysipelas, scarlet fever, measles, small-pox, etc. In what that poison consists we know not; whether it be due to the passage from body to body of living particles of germinal matter, descended from the germinal matter of organism itself, or from the growth and multiplication of vegetable organism, or the result of mephitic vapor, or noxious gas, derived from decaying matter: of this we are ignorant; but we do know that certain effects follow its development in the human system, and that this poison, when once developed, has the power, as a general rule, of reproducing itself. Under this class of diseases I would venture to ask for phlegmonous angina, or quinsy, a place. I believe its constitutional origin can hardly be doubted, when we calmly reflect upon the manner of its invasion; the general lassitude which precedes an attack, the pains in the head and limbs, the alternate chills and flushes of heat, the loss of appetite, the coated tongue, the quick pulse, the high-colored urine, and constipated bowels—symptoms developed before the amygdaloid glands give evidence that *there* is the force of the attack to be expended—all point with unmistakable exactitude to the constitutional and zymotic character of the disease; and, in truth, until the constriction and dryness of the throat, the injection of the fauces, and the enlarging tonsils, reveal the secret, he would be a bold practitioner indeed who would dare to say from the symptoms present that his patient was not going to have a typhoid fever, diphtheria, ague, or some other zymotic affection. Classing, then, with your permission, phlegmonous angina among the zymotic diseases, we will briefly sketch the theory of the use of the sulphites.

From his memoir, it seems, that accepting the zymotic theory as true, Guiseppe Polli, of Milan, noticing the power which sulphurous acid possessed in arresting vinous fermentation, was led to believe that the same agent would arrest catalytic action in the human system. Aware, however, of the danger of introducing sulphurous acid into the animal economy, he conceived the idea of uniting the acid with an earthy base, and thus securing a salt, with which he might safely commence his experiments. The results of his labors are historical, and being no doubt familiar to each of you, I will not trespass upon the time of the Society by detailing them here. I think, however, that his experiment of injecting a dog with the nasal discharge from a glandered horse—having first taken the precaution of giving the dog a couple of drachms of bisulphite sodæ daily for several days before the operation—should give us a hint in the therapeutical use of the sulphites, of which we would do well to avail ourselves when possible. In these experiments Dr. Polli found that the action of the sulphites was quite as decided as the sulphurous acid itself in checking fermentation, and established therefrom two great facts. First, that sulphites, as such, are carried into the circulation; and secondly, that the presence of these salts in the liquids and solids of the body retards putrefactive fermentation for a lengthened period.

Notwithstanding the assertion of the celebrated Claude Bernard, that "it is impossible to neutralize these ferments in the living organism," because, he says, "it would be necessary to interfere with the character of the blood to such an extent that it would be no longer capable of maintaining life," Professor Polli next tested the truth of his discovery upon the human organism, and invariably with a satisfactory result, but modestly adds, "To the test of clinical investigation, and clinical result, I leave the issue of my discovery;" and further on, "I wait for the verdict of the clinical students of Europe." His appeal was not long in being answered, for he states that since he published his first memoir, in 1861, he has had no less than 158 papers published in answer to his call—all, or nearly all, confirmatory of his observations. Upon reading the first mention, to me, of Dr. Polli's discovery, published by Dr. De Ricci, of Dublin, in the *Dublin Quarterly Journal*, November, 1863, I determined to test the matter for myself. Unfortunately, at that time no bisulphites were to be had upon the Pacific coast, and it was not until the spring of 1866 that I was able to procure a pound of the bisulphite of soda. My note-book then informs me that I tried this salt in several cases of scarlet fever, giving ten to fifteen grains every three or four hours, without any well-marked advantage over my former treatment by quinine and iron. Again, I find a case of pyemia, in which I gave my patient twenty grains every four hours, without mitigating the disease in any respect. This result I rather anticipated, as the case was not seen until several of the joints contained pus. I find myself next employing the sulphites in the treatment of some cases of typhoid fever; still I was not aware of any striking result that followed, except that they had the power of diminishing the extreme fetor of the alvine evacuations. Many other articles in the materia medica possessing the same power, my faith in the therapeutical value of the sulphites became very lukewarm, and I prescribed them but seldom. In 1868 we had an epidemic of a species of influenza, accompanied by a remarkable prevalence of anginose affections, especially acute tonsillitis, terminating in abscess. Believing then, as I do now, that tonsillitis is but the local effect of a general poison, and a remnant of faith remaining in the anti-catalytic power of the sulphites, I determined to give them one or more trials, but in a different manner than heretofore.

My idea was now to push them to saturation, as we do other alkalies in the treatment of another poison, rheumatic fever, and get their constitutional effect, if possible, before the zymotic poison had full possession of the system. It so happened that I was the first patient upon whom my primary experiment was to be made. In April, 1868, I was seized with the usual symptoms of impending disease—lassitude, chilliness alternating with flushes of heat, coated tongue, pain in limbs, etc. Soon the dryness and constriction of the throat were perceived, with pain on deglutition, and that horrible feeling of a foreign body in the pharynx, which in vain you try to dislodge. I now knew that my old friend, acute tonsillitis, was about to develop itself, as I was a victim to such attacks, and had almost made up my mind to put in my regular two weeks of sickness, like a philosopher. Upon examining my throat, I noticed both tonsils swollen, their mucous crypts filled with exudation, the velum palati congested, the back of the pharynx a vivid red color, and dry. I dreaded the suffering appertaining to a tonsillitic abscess, and finally came to the conclusion, that with my ideas upon the zymotic origin of the disease, mine was just the case to ascertain whether there was any virtue in the sulphites or not. Accordingly, I ordered a mixture, containing thirty grains of bisulphite of soda to each dose of a table-spoonful, which was to be taken regularly every hour. I commenced at 12 noon; at 8 P. M. I felt decidedly better, and could swallow with but little inconvenience; the tonsillitic exudation had disappeared, and the vivid redness of the velum and pharynx was subsiding. I continued the medicine regularly throughout the night, and at eight next morning almost all traces of the disease had vanished. This result was so striking, and so exceedingly gratifying to me, that I determined to pursue my investigations further.

Two days afterward, Mr. D. called at my office, complaining of the usual initiatory symptoms of tonsillitis. His tonsils, although swollen and congested, had not as yet given any evidence of exudation upon their surface; he was very feverish, with intense thirst, and some pain upon swallowing. This was his second attack within six months. I prescribed for him thirty grains bisulph. sodæ every hour, which he promised to take faithfully, as I informed him that it was only an experiment I was trying, and that if it failed he would be no worse off than before. Next morning he called again; the fever and thirst had subsided; his tongue was cleaning; the redness of the fauces was fading; and, altogether, he was very much better. That evening he declared himself well, but continued his medicine, to make the cure certain.

A third case presented itself soon after; but one tonsil was swollen, and the initiatory stage was not severe. In this case I thought I might diminish the dose of the medicine, and produce, perhaps, as good results: accordingly, I administered twenty grains every three hours; at night the medicine was omitted; next morning the constriction of the throat set in, the redness extending across the velum palati; as evening approached all the symptoms became aggravated, and finally the case terminated in abscess. Still not fully alive to the necessity of saturating the system to produce a successful result, I treated my fourth case with scruple doses of the drug every three hours, without success —the case terminating in abscess upon the tenth day. I was now fully convinced that my first idea was either the true one in this disease, or that the sulphites were worthless, and that both my own and Mr. D.'s case would have terminated in resolution without any treatment. When, therefore, my fifth case presented itself, I ordered thirty grains every hour, day and night. In this case, although the symptoms of acute tonsillitis were as well marked as in either of the others, the result was quite different: in twenty-four hours all signs of the disease had been subdued, and the patient was convalescent. In the following cases the results were the same:

Mrs. McJ.; ailing twelve hours; complains of chills, alternating with fever; pains in the back and limbs, and sore throat. Upon examination the left tonsil was seen engorged; velum palati and back of pharynx very red; great dryness of throat, with pain on swallowing. Ordered thirty grains of bisulph. every hour, day and night; in twenty-four hours all severe symptoms had ceased, and convalescence was established.

Mrs. McD., a resident in the same house, was attacked one week subsequent to the above case. The initiatory symptoms set in with great severity; in twelve hours from the premonitory chill both tonsils were covered with a white exudation, and the pain on deglutition was intense; in addition to the other symptoms, there was engorgement of submaxillary and parotid glands with shooting pains through both ears: in fact, the case closely resembled an attack of diphtheria in its prominent features. I ordered the usual thirty grains of sodæ bisulph. every hour, together with a gargle of the hyposulph. of soda and honey, to relieve the great dryness of the pharynx. In this case, thirty-six hours constant treatment seemed to allay all the worst symptoms of the attack, and in twelve hours more convalescence was established.

Mr. F. has since childhood been the subject of acute tonsillitis, whenever any derangement of his health occurred—the attack almost always ending in abscess. Has tried every known remedy to avert the attack or shorten its duration; has succeeded twice in preventing abscess by constant leeching in the premonitory stage; when that failed, he found that no medication would succeed. In January last he came to me, complaining of his usual symptoms preceding an attack of tonsillitis. I prescribed the sulphite, upon condition that he would take it as ordered, as I considered his case a crucial one. Next morning he called to say that he was no better, but certainly no worse; had taken the medicine regularly until twelve midnight, then fell asleep, and, of course, omitted it. I strongly urged the continuance of the remedy for another twenty-four hours, and then if he was no better I would confess my disappointment; he did so, and the following morning met me in the street, feeling, as he said himself, "All right." He had acknowledged his disbelief in the power of medicine to save him from abscess, as he had taken "bucketsful" without being relieved. He was now satisfied, however, that the bisulphite of soda had the power, and was determined to resort to it again upon the first intimation that his old enemy was preparing for an attack.

I could increase this list by some fourteen additional cases, but as they would be only a repetition of those already enumerated I will not occupy the time of the Society in detailing them. Suffice it to say, that among the fourteen not one terminated in abscess, and not one exceeded forty-eight hours in duration after the commencement of the treatment by the sulphites.

The last cases that I find in my note-book, as treated by the sulphites, are three of diphtheria. One was a child, two years old, to whom I was called last December. She had then been two days ill with what the mother supposed to be an ordinary sore throat. The child appeared cheerful, ate and drank without seeming difficulty or pain. Upon inspection of the throat, I found both tonsils, velum palati and back of pharynx literally covered by a diphtheritic exudation, of a yellowish color and leathery consistence; around the outer margin of the false membrane was a thin, red line, as if separation was about to take place. The examination caused the child to cough, but no stridor was then perceived. The mother said that the only serious symptom the child presented to her was great restlessness at night, with some heat of skin. I ordered eight grains of bisulph. every hour. The following morning both tonsils, velum and pharynx were of a vivid red color, but the exudation had disappeared. The child had slept better, was still cheerful, and drank her milk greedily; her respiration was a little hurried, and her cough a little raucous, which boded no good. I continued the sulphite. Toward night high fever supervened, and it was evident, from her cough, that the false membrane had crept into the larynx. Diphtheritic croup was established, and in twelve hours more the child had breathed her last sigh.

The remaining cases occurred upon Jibboom street—one of the worst localities, if not the very worst, in the city for such a disease to occur.

A. D., aged three years, was seized upon the night of the 23d

January with a severe chill, followed by high fever; next day he appeared to be much better, but his mother noticed that he refused to eat, and had considerable thirst. The same night the fever was very intense, and next morning (25th) the child complained of his throat being sore. I was then sent for. Upon arrival I found the little fellow sitting up in bed, very feverish, but cheerful; his face was flushed, pulse quick, tongue coated, and breath fetid. After some little coaxing, I succeeded in getting a good view of his throat. The examination revealed one-half of the velum pendulum, together with the left tonsil, covered by an exudation exactly similar in appearance to the pedicle left by collodion after the evaporation of the ether. He did not complain of any difficulty or pain on swallowing. His mother had no idea that her child was seriously ill of so fatal a disease as diphtheria; it was therefore necessary for me to impress the fact upon her mind, in order to have my instructions faithfully carried out. I ordered the child a strictly milk diet, and ten grains bisulph. sodæ every hour, day and night. In twenty-four hours of this treatment the pedicle had disappeared from the velum, the fever had ceased, and sleep was easy. A white patch still remained upon the tonsil, which another twenty-four hours treatment sufficed to remove, and the child made an uninterrupted recovery.

A few days afterward I was summoned to see the infant sister of the same boy. Found, upon inspection, that both tonsils were covered by false membrane, extending backward to the pharynx; the velum was free from it. To this infant, fifteen months old, I gave five grains bisulph. sodæ every hour. Thirty-six hours constant treatment removed all trace of exudation, and the child recovered.

These three are the only cases of diphtheria in which I have relied exclusively upon the bisulphite of soda as the curative agent, in addition to the *vis medicatrix naturæ.* The number is too small to base any opinion upon as to the value of the sulphites in this disease. It certainly seemed to possess the power of speedily removing the false membrane, and arresting the disease, or in these particular cases the *vis medicatrix naturæ* worked with unwonted vigor. I am, at all events, encouraged to give the drug a further trial, should any cases of diphtheria present themselves during the year.

I have now fulfilled my promise, and laid before you a series of cases of actual or impending disease, eight of which I have given somewhat in detail; the remaining fourteen being similar in their rapid convalescence, their amplification was not deemed necessary. In all, therefore, I have enumerated twenty-two cases of acute phlegmonous angina, twenty of which were either summarily arrested, or their duration abridged, under the use of the sulphites; that is, unless you consider that the *vis medicatrix naturæ* stepped in methodically and cured these twenty cases, I can ascribe their rapid amelioration to no other cause than the anti-catalytic action of the sulphites. If you concede to me this point, then is my task complete in disproving the sweeping assertion of Trousseau, "That medicine cannot prevail against this disease; that we are powerless to check its progress or abridge its duration." Nay, not content with this, he says, as if to impress the matter fully on his reader's mind, "I repeat, for the third time, that anti-phlogistics, revulsives, topical astringents, and *all* other kinds of treatment, are without power to impede the course of inflammatory sore throat—the duration of which *nothing* can curtail;" and further asserts, "that once inflammatory sore throat has declared itself, it never goes back." There is one other remark of Trousseau's which I will here notice for its great candor. He says, that "there are men who maintain gravely that they can cut short a quinsy in three days," and asks, "Where is the physician of skill sufficient to decide whether a sore throat, which has just made its appearance, is certain to be a quinsy? For my own part," says this physician, "I completely renounce all claim to ability to give a positive opinion under such circumstances, and I doubt whether others are more competent."

As this paper, Mr. President, brings me directly under the charge of maintaining the possibility of cutting short a quinsy in three days, and inability to diagnose the disease, I reply by saying that it is not necessary to diagnose infallibly that the case will be one of quinsy. Suppose the case to be one of membranous sore throat, so often confounded with diphtheria, or acute pharyngitis, or rheumatic sore throat, or any other anginose affection. What does it matter? Is it not sufficient for any physician to know that he has a case of impending disease before him; and if he can by therapeutical, or any other means, arrest the spread of zymosis, and thus cut short the impending disorder, what difference does it make as to the particular portion of the organism in which that zymotic poison would have developed itself? The physician's great triumph consists in his power, first, to prevent, and secondly, to arrest disease in its incipiency, and not wait for hours or days to ascertain whether the *prodromata* of disorder will develop themselves in a typhoid fever or a quinsy. And I assert, as far as the experience of twenty cases will permit me, that phlegmonous angina, or take it generally, a common sore throat, with all the incipient symptoms of acute tonsillitis, can be cut short, or its duration abridged, by the sulphites, and especially the bisulphite of soda. But in order to obtain this result, it is, in my opinion, necessary: First, to saturate the system as rapidly as possible with the salt. Secondly, that any dose under thirty grains an hour is liable to fail in this effect. Thirdly, that the bisulphite of soda is the most reliable salt for that purpose, as both the hyposulphite of soda, and the bisulphite of magnesia, are very prone to purge, which would defeat the object intended. Fourthly, all acids, either in the shape of drinks or gargles, must be strictly prohibited, as they decompose the salt, and render it inert. Lastly, the treatment should be commenced at the very earliest moment possible, and persisted in steadily, day and night, for at least forty-eight hours. If at the end of that time, the urine and the perspiration giving evidence of the thorough impregnation of the system with the salt, the disease is not arrested, I believe further medication to be useless, and the disease will run its usual course, to end in abscess.

I will now have the honor of submitting, for your deliberation and discussion, these remarks upon the value of the sulphites as a therapeutical agent in disease. Although I am afraid that I have fallen very far short in the object I had in view, I will trust in your kind indulgence, and ask your patient consideration for the propositions advanced in this paper:

First, that acute tonsillitis, or phlegmonous angina, and the membranous angina of Trousseau—diseases as closely allied as possible—are zymotic in character, and as much constitutional diseases as scarlet fever or diphtheria. Secondly, that, as zymotic in character, they are amenable to treatment by anti-catalytics, when properly and persistently administered. Thirdly, that in the bisulphite of soda we have a safe and efficient agent in the treatment of such diseases, and presumptive proof that when administered early in all diseases of like zymotic character, it will prove as efficacious as in the diseases under consideration this evening. And lastly, that when given under the circumstances proper for their administration, the sulphites are perfectly harmless in their effect, seldom producing vomiting or disorder of the bowels; and if at times they seem inefficient to arrest retrograde metamorphosis, they leave the patient in no worse condition than before.

I therefore will ask for your co-operation in giving this drug an extended trial, and am persuaded that you will not refuse your support, where you perceive an honest effort to arrive at a scientific truth, as it is only by the experience of many that a correct deduction can be made, and the value of any discovery ascertained. —*Pacific M. and S. Journal.*

